# MicroRNA-200c Release from Gelatin-Coated 3D-Printed PCL Scaffolds Enhances Bone Regeneration

**DOI:** 10.1021/acsbiomaterials.3c01105

**Published:** 2024-03-26

**Authors:** Matthew T. Remy, Chawin Upara, Qiong J. Ding, Jacob M. Miszuk, Hongli Sun, Liu Hong

**Affiliations:** † Iowa Institute for Oral Health Research, College of Dentistry, The University of Iowa, Iowa City, Iowa 52242, United States; ‡ Roy J. Carver Department of Biomedical Engineering, College of Engineering, The University of Iowa, Iowa City, Iowa 52242, United States

**Keywords:** miR-200c, gelatin, PCL, bone regeneration, drug delivery

## Abstract

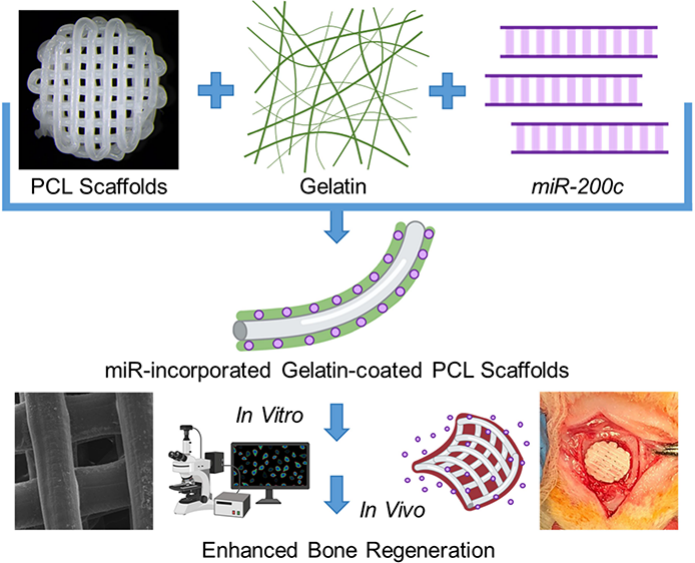

The fabrication of clinically relevant synthetic bone grafts relies on combining multiple biodegradable biomaterials to create a structure that supports the regeneration of defects while delivering osteogenic biomolecules that enhance regeneration. MicroRNA-200c (*miR-200c*) functions as a potent osteoinductive biomolecule to enhance osteogenic differentiation and bone formation; however, synthetic tissue-engineered bone grafts that sustain the delivery of *miR-200c* for bone regeneration have not yet been evaluated. In this study, we created novel, multimaterial, synthetic bone grafts from gelatin-coated 3D-printed polycaprolactone (PCL) scaffolds. We attempted to optimize the release of pDNA encoding *miR-200c* by varying gelatin types, concentrations, and polymer crosslinking materials to improve its functions for bone regeneration. We revealed that by modulating gelatin type, coating material concentration, and polymer crosslinking, we effectively altered the release rates of pDNA encoding *miR-200c,* which promoted osteogenic differentiation *in vitro* and bone regeneration in a critical-sized calvarial bone defect animal model. We also demonstrated that crosslinking the gelatin coatings on the PCL scaffolds with low-concentration glutaraldehyde was biocompatible and increased cell attachment. These results strongly indicate the potential use of gelatin-based systems for pDNA encoding microRNA delivery in gene therapy and further demonstrate the effectiveness of *miR-200c* for enhancing bone regeneration from synthetic bone grafts.

## 1 Introduction

Developing synthetic bone grafts through tissue engineering strategies is essential to overcome natural grafting limitations to treat large bone defects.^1^ Tissue-engineered synthetic bone grafts can be created by combining biomaterials, such as ceramics, natural or synthetic polymers, and regenerative biomolecules. For effective bone regeneration, synthetic grafts not only need to be fabricated using biomaterials that mechanically support the defect as it regenerates but should also provide a mechanism to incorporate osteoinductive biomolecules that stimulate osteogenic differentiation and bone formation.^2^ Recently, multiple osteoinductive microRNAs (miRs) have been identified that regulate osteogenic pathways and play critical roles in enhancing bone regeneration.^3−6^*miR-200c*, a member of the miR-200 family, is a well-known anticancer miR that effectively suppresses many types of cancers, including oral squamous cell carcinoma and osteosarcoma.^7,8^*miR-200c* also effectively increases osteogenic differentiation *in vitro*,^9−12^ and when incorporated into collagen coatings on three-dimensional (3D)-printed bioceramics, *miR-200c* increased bone formation *in vivo* in a critical-sized rat calvarial bone defect model.^13^ As a potent, osteoinductive biomolecule, *miR-200c* effectively enhances bone regeneration for tissue engineering;^4,9−13^ however, restoring large bone defects is a long, complex process that requires prolonged stimulation of osteogenic factors and a delivery system that sustains the release of osteoinductive agents, like *miR-200c*, from synthetic tissue-engineered bone grafts is yet to be developed.

In bone tissue engineering strategies, synthetic polymers with tunable biodegradation rates, including poly(lactic acid) (PLA) and poly(caprolactone) (PCL), have gained appreciative use as alternatives to slow-degrading bioceramics, like hydroxyapatite (HA) and β-tricalcium phosphate (β-TCP).^14−19^ PLA and PCL have previously been utilized in synthetic bone grafts and can be used to fabricate three-dimensional (3D), porous scaffolds via methods like fused deposition modeling.^20^ However, the biodegradation of PLA releases acidic byproducts *in vivo*, which causes an increase in local inflammation and, therefore, has limited its use in bone tissue engineering applications.^21^ Alternatively, PCL, an FDA-approved material used in drug delivery, is increasingly used to fabricate synthetic bone grafts.^22^ However, the hydrophobic nature of PCL results in poor cell attachment. Therefore, PCL must be combined with hydrophilic biomaterials to improve cell attachment, facilitate osseointegration, and promote osteogenesis.^23−26^ Coating synthetic grafts with natural polymers, such as collagen or gelatin, is an effective method to increase scaffold hydrophilicity and mechanical strength and provides a mechanism to release incorporated biomolecules.^27^ By exploiting the hydrophilic and electrostatic properties of gelatin, 3D-printed PCL scaffolds coated with gelatin may function as a delivery system to sustain osteoinductive miR release and enhance bone regeneration from synthetic grafts.

**Figure 1:**
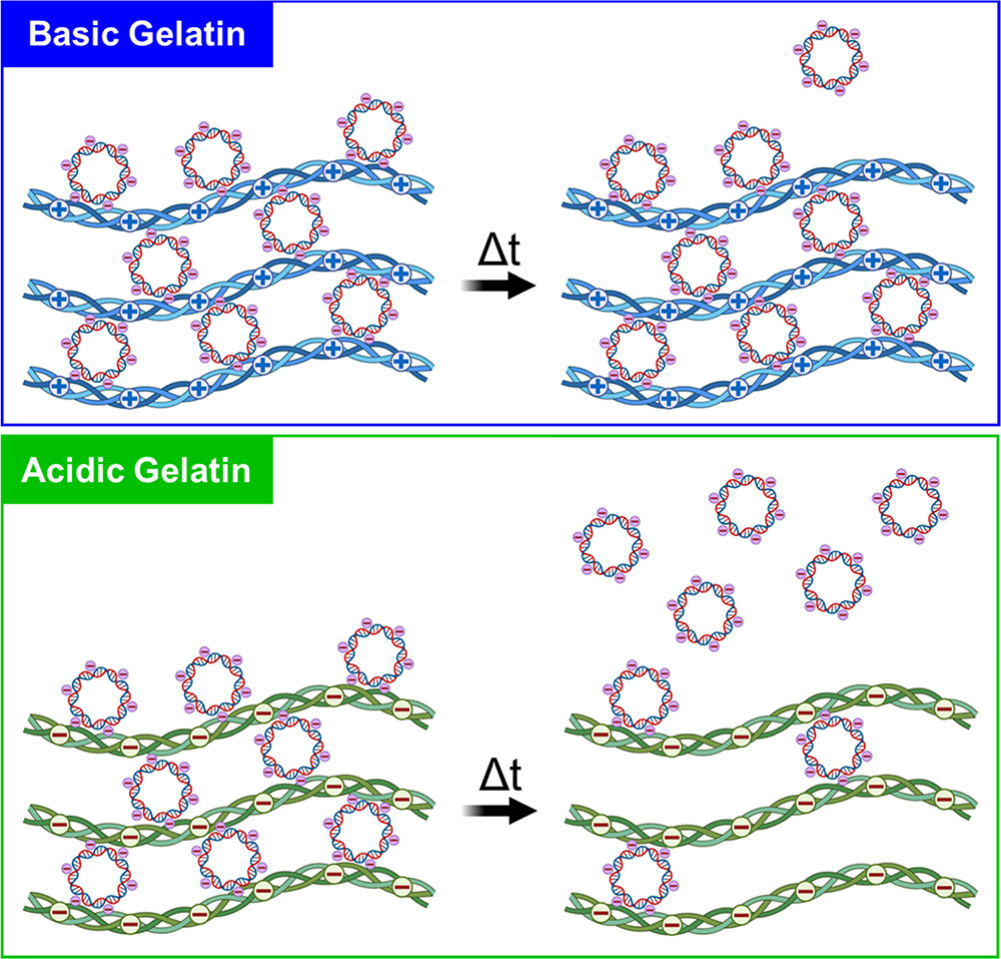
Illustration relating the release of negatively charged pDNA from different gelatin types. In the delivery of negatively charged pDNA (pI = 5) at physiological pH, the similarly charged side chains in the negatively charged gelatin polymer (pI = 5) would likely cause electrostatic repulsion and rapid release of pDNA into the local environment. However, the differences in charge between the positively charged basic gelatin polymer (pI = 9) and the negatively charged pDNA would likely create bonding opportunities that sustain pDNA release over time. Figure created with Biorender.com.

Gelatin, a natural polymer derived from collagen, can be produced through alkaline or acidic treatment processes to create two polymeric systems with differing electrostatic charges. Through alkaline treatment, amide groups on collagen are hydrolyzed, leaving gelatin chains to possess a high density of carboxyl groups, yielding a negatively charged, acidic gelatin with a lower isoelectric point (pI = 5). In the acidic treatment process, however, the amide groups are hardly modified, yielding an alkaline or basic gelatin that possess a more positively charged structure with a higher isoelectric point (pI = 8–9).^28^ Thus, gelatin can be modified to exhibit different isoelectric points that can be exploited for complexation with positively or negatively charged biomolecules.^29^ Prior studies have primarily employed these charge differences to incorporate acidic, negatively charged gelatin hydrogels with positively charged growth factors, including basic fibroblast growth factor (bFGF), vascular endothelial growth factor (VEGF), bone morphogenetic proteins (BMPs), and others.^30−34^ However, positively charged, basic gelatin may have significant application in sustaining the delivery of negatively charged plasmid DNAs (pDNA) encoding osteoinductive biomolecules, such as pDNA encoding miRs, for gene therapy and bone regeneration (Figure 1). Using a gelatin coating system, we aim to sustain the release of osteoinductive *miR-200c* and prolong regenerative signaling from 3D-printed synthetic grafts to promote bone regeneration.

The clinical translation of synthetic bone grafts to date has been hindered by factors such as inappropriate scaffold materials, inadequate scaffold fabrication strategies, and the reliance on inefficient delivery systems. Building upon these prior studies, this work investigates the design of a novel multimaterial synthetic bone graft to sustain release of osteoinductive miRs from natural polymer-coated, 3D-printed constructs. Leveraging the electrostatic properties of gelatin, this work evaluates how differences in gelatin type, coating material, and crosslinking concentration influence the release of pDNA encoding osteoinductive *miR-200c* from 3D-printed PCL scaffolds for bone regeneration. These findings advance our understanding of how pDNA encoding miR release from polymer-coated synthetic grafts can be modulated to sustain regenerative signaling. By using a multimaterial, 3D-printing approach, our gelatin-coated PCL scaffolds overcome the limitations of current, single-material synthetic bone grafts. Our novel strategy that employs both synthetic and natural polymers to sustain the delivery of potent osteoinductive miRs is needed to significantly enhance bone regeneration from tissue-engineered bone substitutes and advance the design of synthetic bone grafts toward effective clinical use for treating large bone defects.

## 2 Materials and Methods

### 2.1 Fabrication of 3D-Printed PCL Scaffolds

PCL scaffolds were 3D-printed by using fused deposition modeling (FDM). The PCL filament was commercially purchased (Facilan PCL 100 Filament; 3D4Makers, Haarlem, Netherlands) and threaded into the FDM syringe component on a Regemat 3D bioprinter device (Regemat 3D, Granada, Spain) for printing of the PCL scaffolds. PCL scaffolds were 3D-printed using printing parameters and dimensions found in Table 1.

**Table 1:** Parameters Used to 3D-Print PCL Scaffolds

printing parameters		scaffold dimensional properties	
infill speed (mm/s)	2.05	height (mm)	2.00
flow speed (mm/s)	0.55	diameter (mm)	9.00
travel speed (mm/s)	50.00	pore size (mm)	0.60
printing temperature (°C)	65.00	layer height (mm)	0.35

### 2.2 Development and Characterization of Coated PCL Scaffolds Incorporating miR-200c

All coating solutions and crosslinking materials were commercially purchased: collagen (Coll) solution (Corning, MA, USA) and sponges (Zimmer Biomet, Warsaw, IN); acidic gelatin (AG) and basic gelatin (BG) powder (Sigma-Aldrich, MO, USA), and sponges (Nitta Gelatin, Osaka, Japan); glutaraldehyde (GTA) (Thermo Fisher Scientific, MA, USA); genipin (GNP, Thermo Fisher Scientific, MA, USA); and glycine (Research Products International, IL, USA). Scaffold treatment groups for different experiments within this study were created to test low [L], medium [M], and high [H] concentrations for each coating material and crosslinking agent based on standard concentrations reported in the literature.^35−42^ Treatment group conditions included are as follows: (A) coating materials (Coll, AG, BG): [L]: 1 mg/mL, [M]: 3 mg/mL, [H] 8 mg/mL; (B) crosslinking agents: (1) GTA: [L]: 0.01 v/v%, [M]: 0.05 v/v%, [H]: 0.10 v/v%; (2) GNP: [L]: 0.01 wt %, [M]: 0.05 wt %, [H]: 0.10 wt %. All material preparation and scaffold coating procedures occurred in a biological safety cabinet under sterile conditions.

**Figure 2:**
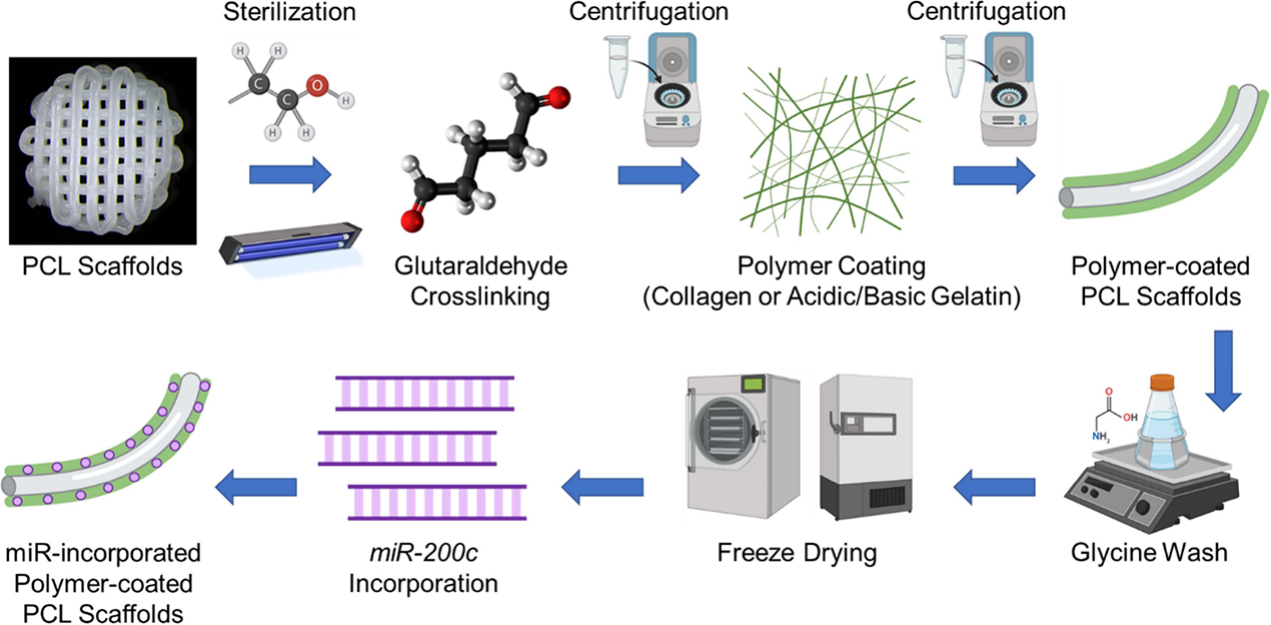
Scaffold fabrication and polymer-coating process to produce pDNA encoding *miR-200c*-incorporated polymer-coated PCL scaffolds. Figure created with Biorender.com.

Prior to coating, the 3D-printed PCL scaffolds were sterilized by using a series of washes with 70% ethanol followed by subsequent washes with phosphate-buffered saline (PBS) and exposure to ultraviolet light for 15 min. After sterilization, 3D-printed PCL scaffolds were first dip-coated in crosslinking solution for 30 s, and then, the scaffolds were placed in a vacuum-sealed tube, where the air was removed via syringe for 30 s to seal the crosslinker coating solution onto the PCL scaffolds. Excess crosslinking solution was then removed from the scaffolds via centrifugation at 800 rpm for 15 s. Once the crosslinker coat was added, the coated scaffolds were then dip-coated in either collagen or gelatin (acidic or basic) solution for 30 s, followed again by vacuum-sealing of the materials to the scaffolds and subsequent centrifugation of excess solution. Scaffolds that did not require crosslinking were not exposed to crosslinker solution and were only dip-coated using either collagen or gelatin (acidic or basic) solution. After the coating process, scaffolds were individually placed into wells in a 48-well plate containing glycine solution [100 mM] to quench, neutralize, and inactivate any residual aldehyde groups from glutaraldehyde crosslinking.^43−46^ The scaffolds were agitated in a glycine solution on a shaker for 15 min at room temperature. After the glycine wash, the coated scaffolds were individually placed into new wells on a 48-well tissue culture plate and allowed to dry for 15 min at room temperature prior to being frozen at −80 °C overnight and lyophilized for 6 h using a freeze-drying machine (Labconco, MO, USA). Once the coated PCL scaffolds were freeze-dried, solution containing pDNA encoding *miR-200c* was added onto the coated PCL scaffolds and the pDNA solution was allowed to incorporate into the coated PCL scaffolds for 30 m at 4 °C prior to being frozen at −80 °C overnight and lyophilized for 6 h. *miR-200c*-incorporated, non-crosslinked and GTA-crosslinked, polymer-coated PCL scaffolds, referred to as PCL/(Coll or AG or BG) [*miR-200c*] and PCL/(Coll or AG or BG)/GTA [*miR-200c*], were fabricated in this manner for all experiments related to this study, utilizing an array of low [L], medium [M], and high [H] coating material or crosslinking agent concentrations as previously described. Figure 2 illustrates the fabrication process used to create our miR-incorporated, polymer-coated PCL scaffolds. Coating thickness and distribution on the surface of the 3D-printed PCL scaffolds were characterized and visualized via field-emission scanning electron microscopy (FE-SEM; Hitachi S-4800, Tokyo, Japan) and Coomassie blue staining (Thermo Fisher Scientific, MA, USA) following manufacturer’s protocols. Scaffold dimensions and porosity were measured using a caliper, ImageJ software (NIH, MD, USA), and liquid displacement methods.

### 2.3 Release of pDNA Encoding *miR-200c* from Polymer-Coated PCL Scaffolds

pDNA release from non-crosslinked and crosslinked collagen- or gelatin-coated PCL scaffolds was quantified across different time points to evaluate the different release profiles associated with each coating and crosslinker combination. Coated PCL scaffolds were also compared to commercially available sponges to evaluate the efficiency of the PCL coating system in relation to that of commercial products. To study pDNA release, PCL scaffolds were coated and crosslinked via the fabrication system described using an array of polymer coating and crosslinker concentration combinations. All scaffolds for the release study were incorporated with 1 μg of pDNA suspended in 20 μL of PBS. The volume of the pDNA solution is entirely absorbed by the scaffolds. For the release study, pDNA-incorporated, coated PCL scaffolds, or commercial collagen or gelatin sponges, were individually placed into wells of a 48-well tissue culture plate containing 500 μL of PBS and placed on a shaker to continuously shake at 150 rpm and 4 °C for the duration of each study. To quantify released pDNA, at each time point, half of the PBS solution was removed for double-stranded DNA analysis, and fresh PBS solution was added back to the well. The concentration of pDNA released from the coated PCL scaffolds was quantified using a Qubit dsDNA HS assay kit (Invitrogen, MA, USA) at distinct time points following the manufacturer’s protocols. pDNA concentration for each scaffold was measured in triplicate.

### 2.4 Evaluating Biocompatibility of Crosslinked Collagen- or Gelatin-Coated PCL Scaffolds

During the study, we observed that a high GTA concentration of [H] led to hydrogel clotting within the porous structure of 3D-printed scaffolds, significantly affecting pore size and cell migration. Consequently, we chose to use a gelatin coating with a medium GTA concentration [M] for further testing of scaffold toxicity functionality incorporating *miR-200c* for osteogenic function assessment both *in vitro* and *in vivo*. The toxicity of GTA as a crosslinking agent on the viability of human preosteoblasts, embryonic palatal mesenchymal (HEPM; ATCC, VA, USA) cells was assessed via an MTT cell viability assay kit (Biotium, CA, USA) according to the manufacturer’s protocol. HEPM cells were seeded onto PCL scaffolds (5 × 10^5^ cells/scaffold) coated with collagen, acidic gelatin, or basic gelatin, with or without a GTA crosslinker at 0.05 v/v%. The HEPM cells were cultured on the coated PCL scaffolds in a 48-well tissue culture plate in Dulbecco’s modified Eagle’s medium (DMEM) supplemented with 10% fetal bovine serum and promocin (100 μg/mL; Life Technologies, NY, USA) at 37 °C and 5% CO_2_ for 48 h, after which the scaffolds were removed and placed into new wells in a 48-well tissue culture plate, and MTT solution was added to the scaffold-containing wells. MTT absorbance was measured and normalized to the HEPM cells seeded on non-GTA crosslinked, coated PCL scaffolds following the manufacturer’s protocol on a spectrophotometer (SpectraMax iD2; Molecular Devices, CA, USA) at 570 nm, with background absorbance set to 630 nm.

### 2.5 Microscopic Imaging of Cell-Seeded Collagen- or Gelatin-Coated PCL Scaffolds

HEPM cell morphology and distribution on coated PCL scaffolds were visualized by using confocal microscopy (LSM 710; Zeiss, CA, USA) and DAPI (Sigma-Aldrich, MO, USA) staining methods. Briefly, HEPM cells were seeded onto non-crosslinked and crosslinked, collagen- or gelatin-coated PCL scaffolds (5 × 10^5^ cells/scaffold) and cultured in a 48-well tissue culture plate in DMEM supplemented with 10% fetal bovine serum and 100 μg/mL promocin at 37 °C and 5% CO_2_ for 48 h. After 48 h in culture, the HEPM cell-seeded PCL scaffolds were fixed using 4% paraformaldehyde for 15 min, followed by two washes with PBS. The fixed scaffolds were stained with nuclear DAPI (excitation/emission: 358/461 nm) and fluorescein phalloidin (excitation/emission: 496/516 nm) solution following manufacturer’s protocols (Thermo Fisher Scientific, MA, USA). The fluorescently stained cells were imaged using a confocal microscope at 10× magnification, and representative z-stack and 3D images were collected to illustrate HEPM cell distribution at the surface and within the coated scaffolds.

### 2.6 Analysis of pDNA Encoding *miR-200c* Transfection Efficiency and Osteogenic Biomarker Production from Preosteoblasts Seeded on PCL Scaffolds

To evaluate differences in *miR-200c* transfection efficiency of the differently coated PCL scaffolds, HEPM cells were first seeded onto non-crosslinked and GTA (0.05 v/v%)-crosslinked acidic or basic gelatin-coated PCL scaffolds (5 × 10^5^ cells/scaffold) incorporated with *miR-200c* [5 μg] and cultured for 3 days according to previous studies.^10^*miR-200c* transfection efficiency induced by the release of *miR-200c* from the different gelatin coatings with and without GTA crosslinking on the PCL scaffolds was assessed via qRT-PCR, using methods previously described (performed using technical triplicates).^9,13,47^ Briefly, total cellular RNA from HEPM cells cultured on the coated PCL scaffolds was extracted using a miRNeasy Micro Kit (Qiagen, CA, USA). The concentration and purity of RNA were quantified using spectrophotometry (NanoDrop One Microvolume UV–vis; Thermo Fisher Scientific, MA, USA). To measure *miR-200c* expression, the Mir-X miRNA first strand synthesis kit (Takara Bio, Inc., Kusatsu, Japan) and TB Green Premix Ex *Taq*II (Takara Bio, Inc., Kusatsu, Japan) were used and normalized to *U6* via a comparative Ct (ΔΔCt) method.

To assess the influence of *miR-200c* release on osteogenic biomarker expression, the mRNAs of osteogenic biomarkers, including runt-related transcription factor 2 (*RUNX2*), osteocalcin (*OCN*), and alkaline phosphatase (*ALP*), were evaluated by using qRT-PCR. HEPM cell-seeded PCL scaffolds incorporated with *miR-200c* [5 μg] were cultured for 3 days, as in the *miR-200c* transfection study. After 3 days of culture, total cellular RNA from HEPM cells seeded onto the coated PCL scaffolds was extracted using the methods previously described. osteogenic biomarker expression was evaluated on a CFX Connect instrument (Bio-Rad, CA, USA) using the SYBR Premix Ex Tag II Kit (Takara Bio, Inc., Kusatsu, Japan). Gene expression was calculated and compared with *GAPDH* via a comparative Ct (ΔΔCt) method. Primer sequences for the gene expression studies are listed in Table 2. In a supplemental study, HEPM cells were seeded onto non-crosslinked basic gelatin and GTA-crosslinked acidic or basic gelatin-coated PCL scaffold (4 × 10^5^ cells/scaffold) incorporated plasmid encoding *miR-200c* [5 μg] as previously described. The constructs were cultured for 14 days in DMEM medium supplemented with 5 mM β-glycerophosphate and 50 μM l-ascorbic acid at 37 °C and 5% CO_2_. We stained the scaffolds after 14 days using an ALP staining kit (Sigma-Aldrich) following the manufacturer’s instructions. Images of the scaffolds were taken at 15× magnification, and the average intensity of the stain was quantified using the gray value measurement in ImageJ.

**Table 2:** Primer Sequences Used for In Vitro qRT-PCR Analyses

**gene**	**forward primer (5′ → 3′)**	**reverse primer (5′ → 3′)**
*U6*	GTGCTCGCTTCGGCAGCA	CAAAATATGGAACGCTTC
*RUNX2*	TGGTTACTGTCATGGCGGGTA	TCTCAGATCGTTGAACCTTGCTA
*OCN*	GGCGCTACCTGTATCAATGG	GTGGTCAGCCAACTCGTCA
*ALP*	ACCACCACGAGAGTGAACCA	CGTTGTCTGAGTACCAGTCCC
*GAPDH*	CACCATGGAGAAGGC	TGCCAGTGAGCTTCC

### 2.7 Critical-Sized Calvarial Bone Defect Model—Surgical Preparation and Animal Care

All *in vivo* animal experiments were performed with the approval of the Office of Institutional Animal Care and Use Committee (IACUC) at the University of Iowa. All biological agents and coated PCL scaffolds were prepared and implanted into 12-week-old male Sprague–Dawley rats (Charles River Laboratories, MA, USA) under sterile conditions in a surgical environment. Under general anesthesia using ketamine/xylazine, a 9 mm diameter full thickness defect was generated on the rat parietal bones following previously described surgical protocols.^13^ A total of four groups of treated scaffolds were implanted into critical-sized defects in the rat calvaria to observe the regenerative effects of the *miR-200c*-incorporated GTA (0.05 v/v%)-crosslinked acidic gelatin (AG) or basic gelatin (BG)-coated PCL scaffolds, including: (1) PCL/AG/GTA [1 μg *miR-200c*], (2) PCL/BG/GTA [1 μg *miR-200c*], (3) PCL/AG/GTA [10 μg *miR-200c*], (4) PCL/BG/GTA [10 μg *miR-200c*]. The doses of *miR-200c* were selected based on previous studies.^10^ Each animal received one treated PCL implant, and each treatment condition had 3–7 animals per group. Rats were euthanized after 6-weeks, and the implanted scaffolds and surrounding bone tissues were harvested. Harvested tissues were first rinsed in PBS and then fixed in 4% paraformaldehyde for 24 h.

### 2.8 MicroCT Imaging and Quantitative Analysis of Bone Regeneration in Calvarial Defects

Microcomputed tomographic (μCT) imaging was performed to evaluate new bone formation within the critical-sized calvarial defect space. Images of the PCL implants in the calvarial defect space were scanned and reconstructed via a μCT scanner with Bruker software (Skyscan model 1272; Bruker, Kontich, Belgium) using the following acquisition parameters: 80 kV, 125 μA, 0.6 rotation step, 1 mm Al filter, and 18 μm pixel size. Dragonfly 2021.3 software (Object Research Systems, Montreal, Canada) was used to analyze bone formation in calvarial defects. Briefly, reconstructed calvarial images were loaded into Dragonfly, and using grayscale intensity thresholding, low-density soft tissues were removed and a data set including all dense bone materials was created. After thresholding, a 6 mm diameter, cylindrical region of interest (ROI) was placed in the calvarial defect space to emulate the implanted scaffold and evaluate bone growth in the central portion of the defect region. Bone formation in the defects was collected as a volume percentage of dense bone data set values within the defect ROI. Bone volume percentage data were collected in this manner, using the same thresholds across all samples, and representative 2D and 3D images were acquired to visualize the differences in bone formation within the defects containing differently treated PCL implants.

### 2.9 Histological Evaluation of *In Vivo* Bone Formation in Calvarial Defects

The explanted calvarial defects were decalcified for 8 h using a decalcification solution (Decalcifying Solution-Lite; Sigma-Aldrich, MO, USA) and cut in half. The decalcified samples were then cleared with xylene and embedded in paraffin. The entire embedded sample, which included the defect with the coated PCL implant and surrounding calvarial tissue, was cut into 7 μm coronal sections and stained with hematoxylin and eosin (H&E) and Heidenhain’s azan trichrome stain. Representative sections were selected for staining at distinct intervals throughout the sample, starting from the middle of the sample and working outward at an interval sampling distance of 0.5 mm (*n* = 5). Corresponding images of the stained tissues were taken using a light microscope (Nikon Instruments, Inc., NY, USA) to examine the bone formation in the critical-sized calvarial defects occurring from the differently treated PCL scaffold implants and the integration of the PCL implants with the native bone tissues.

### 2.10 Statistical Analysis

Descriptive statistics were conducted for both *in vitro* and *in vivo* investigations. A one-way analysis of variance (ANOVA) with post hoc Tukey’s honestly significant difference (HSD) test was used to determine whether there were significant differences between treatment groups for the in vitro *miR-200c* transfection, osteogenic biomarker, and biocompatibility studies. For the in vivo study, a one-way ANOVA with post hoc Tukey’s HSD test was utilized to evaluate whether there were significant differences in bone formation across both the 1 and 10 μg treatment groups, and a Student’s *t* test was used to assess differences in bone formation between acidic and basic gelatin-coated PCL within the 1 or 10 μg treatment groups. The Shapiro–Wilks test was also applied to verify the assumption of normality. All statistical tests completed for the in vitro and in vivo quantifications used a significance level of 0.05, and each graphic depicts the mean values and associated standard deviations. Statistical analyses and associated figures were created via GraphPad Prism (version 8.1.2.; GraphPad Software, Inc., CA, USA).

## 3 Results

### 3.1 Fabrication and Characterization of Gelatin-Coated 3D-Printed PCL Scaffolds

**Figure 3:**
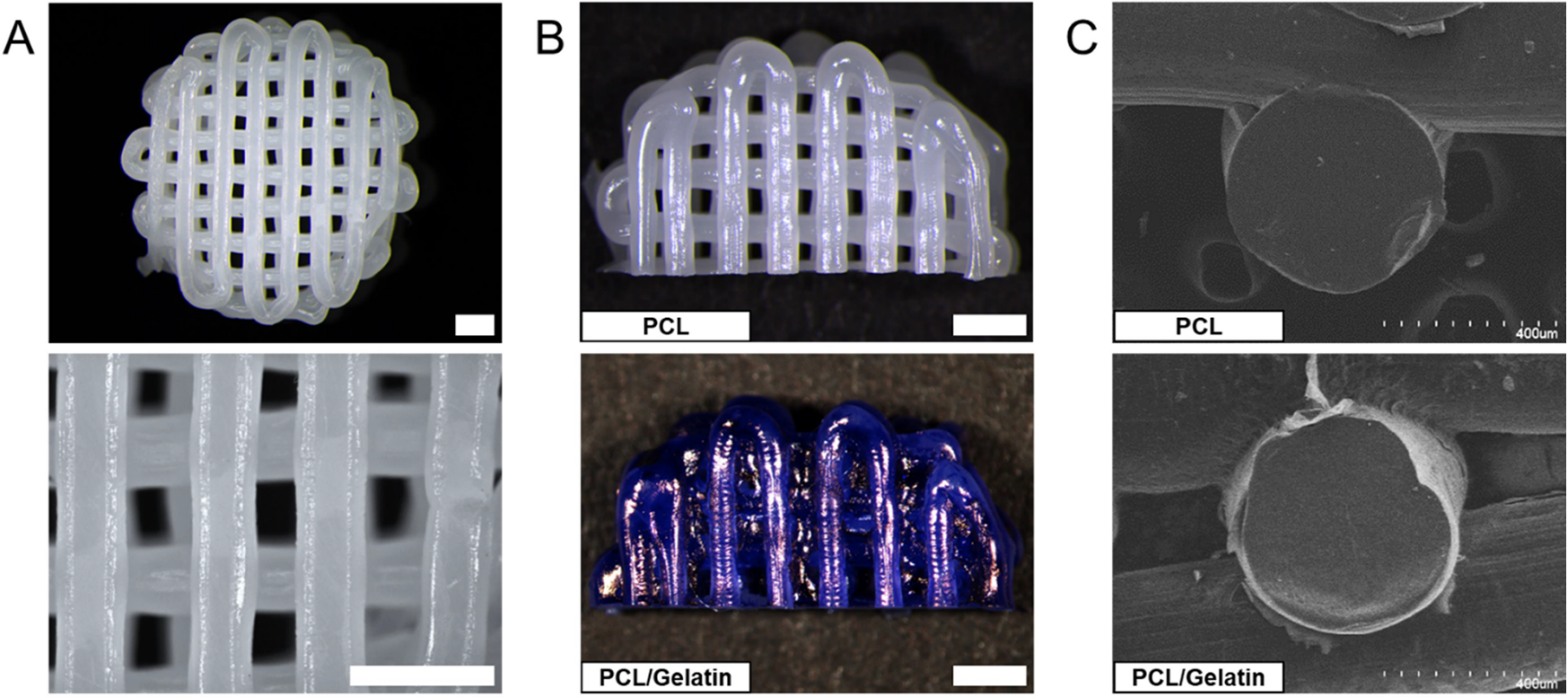
Visualization of polymer coatings on 3D-printed PCL scaffolds. (A) Images of PCL scaffolds 3D-printed using fused deposition modeling. (B) Images of noncoated and gelatin-coated PCL scaffolds stained with Coomassie blue protein stain. (C) SEM images of PCL scaffold filament cross-sectionally cut to display the absence or presence of gelatin coating. Scale bars: 1 mm.

PCL scaffolds were 3D-printed to contain well-defined, interconnected porous channels using fused deposition modeling (FDM) (Figure 3A). The 3D-printed PCL scaffolds were evaluated for mean pore size, porosity, and other dimensional parameters, and these values are reported in Table 3. The average overall diameter of the 3D-printed PCL scaffolds was 8.67 mm with an average height of 2.16 mm. The PCL scaffolds also displayed an average filament diameter and pore size of 500 and 450 μm, respectively, and maintained an average porosity of 55.75% (Figure 3A). The 3D-printed PCL scaffolds were then coated with gelatin using the coating procedures previously described and stained with Coomassie blue protein staining to evaluate and visualize the attachment of gelatin to the PCL scaffold surface (Figure 3B). Using the Coomassie blue protein stain, we found that the gelatin coating demonstrated a homogeneous distribution across the surface of the 3D-printed PCL scaffolds. SEM imaging was then used to further evaluate and visualize the gelatin coatings on the 3D-printed PCL scaffolds under higher magnification. Using SEM imaging, we again found that the coating process produced a thin, homogeneously distributed layer of gelatin across the surface of the 3D-printed PCL scaffolds. We then cut the gelatin-coated PCL scaffold cross-sectionally to observe the coating distribution on a PCL filament and found that the gelatin coating process produced a thin layer of gelatin on the PCL filament with an average coating thickness of 17.52 μm (Figure 3C).

**Table 3:** Dimensional Parameters of the 3D-Printed Polymer-Coated PCL Scaffolds

bulk scaffold dimensions	mean (SD)	minute scaffold dimensions	mean (SD)
scaffold diameter (mm)	8.67 (0.20)	filament diameter (mm)	0.50 (0.02)
scaffold thickness (mm)	2.16 (0.11)	pore diameter (mm)	0.45 (0.10)
scaffold porosity (%)	55.75 (10.88)	coating thickness (μm)	17.52 (5.23)

### 3.2 Coating Material and Crosslinker Concentration Influence pDNA Release from Coated 3D-Printed PCL Scaffolds

**Figure 4:**
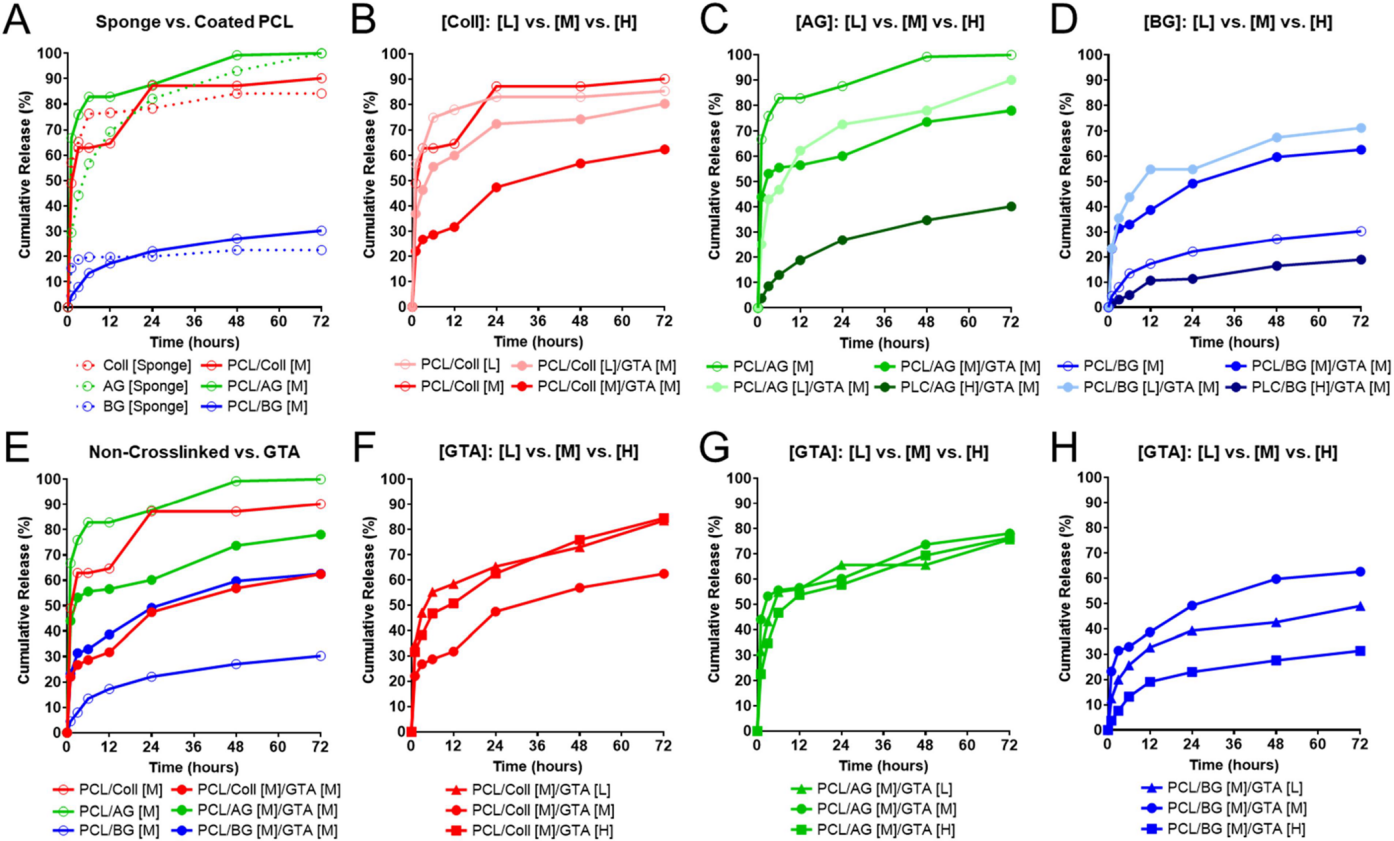
PCL scaffold-coated polymeric material, concentration, and the crosslinking agent concentration influence the release of pDNA. (A) Comparison of pDNA profiles released from sponges of collagen (Coll), acidic gelatin (AG), or basic gelatin (BG) vs Coll-, AG-, or BG-coated PCL scaffolds without GTA crosslinking. (B–D) Profiles of pDNA released from PCL scaffolds coated with either Coll (B), AG (C), or BG (D) and with or without GTA crosslinking. (E) Profiles of pDNA released from non-crosslinked and GTA-crosslinked Coll-, AG-, or BG-coated PCL scaffolds. (F–H) Profiles of pDNA released from GTA-crosslinked PCL scaffolds coated with Coll (F), AG (G), or BG (H) with different GTA concentrations.

3D-printed PCL scaffolds were coated with either collagen, acidic, or basic gelatin incorporating negatively charged pDNA molecules, and pDNA release was evaluated over time. First, we compared our polymer-coated PCL scaffolds to commercially available collagen, acidic, and basic gelatin sponges (Figure 4A). We observed a distinct difference in pDNA release profiles between the three different polymer types, where the collagen and acidic gelatin materials rapidly released incorporated pDNA over 72 h, while the basic gelatin materials displayed a slowed and sustained release profile over 72 h. We also observed that the polymer-coated PCL scaffolds demonstrated similar release profiles to their commercial sponge counterparts (i.e., collagen-coated PCL vs collagen sponge), illustrating that the polymer-coated PCL scaffolds experience burst or sustained release mechanisms in a manner analogous to commercially available sponges.

We then evaluated how modulating polymer coating material concentration influenced pDNA release among the collagen-, acidic-, or basic gelatin-coated PCL scaffolds with or without GTA crosslinking (Figure 4B–D). In the collagen-coated PCL scaffolds (Figure 4B) and acidic gelatin-coated PCL scaffolds (Figure 4C), we found that cumulative pDNA release was reduced throughout the 72 h observation period when the coating materials were crosslinked with GTA in comparison to non-crosslinked coatings at the same polymer coating material concentration. However, for the basic gelatin-coated PCL scaffolds (Figure 4D), we observed an increase in pDNA release when the basic gelatin-coated PCL scaffolds were crosslinked with GTA in comparison to non-crosslinked basic gelatin-coated PCL at the same basic gelatin-coating concentration. However, the level of cumulative release for the basic gelatin-coated PCL scaffolds was less than the collagen- or acidic gelatin-coated PCL scaffolds at the same coating material concentration, regardless of GTA crosslinking. Furthermore, we observed that in each coating material type (collagen, acidic gelatin, or basic gelatin), increasing the polymer coating material concentration from low [L] to medium [M] and high [H] caused a decrease in cumulative pDNA release over the 72 h observation period.

After evaluating the influence of polymer coating material concentration on pDNA release, we then assessed how modulating GTA crosslinking altered pDNA release profiles (Figure 4E–H). Figure 4E summarizes the influence of GTA crosslinking on the release of pDNA from collagen-, acidic gelatin-, or basic gelatin-coated PCL scaffolds. We found that crosslinking with GTA decreased cumulative pDNA release in the collagen- and acidic gelatin-coated PCL scaffolds, while GTA crosslinking of basic gelatin-coated PCL scaffolds demonstrated the opposite effect and increased cumulative pDNA release to an extent comparable to GTA-crosslinked collagen-coated PCL. In the collagen-coated PCL scaffolds (Figure 4F) and the acidic gelatin-coated PCL scaffolds (Figure 4G), increasing the GTA crosslinker concentration had little influence on the cumulative release of incorporated pDNA. However, for the basic gelatin-coated PCL scaffolds (Figure 4H), the concentration of GTA crosslinker had a variable effect on cumulative pDNA release, where basic gelatin coatings crosslinked with high concentrations of GTA demonstrated a reduction in cumulative pDNA release in comparison to basic gelatin coatings crosslinked with low and medium GTA concentrations.

### 3.3 Polymer Coating and Crosslinking on 3D-Printed PCL Scaffolds Are Highly Biocompatible

**Figure 5:**
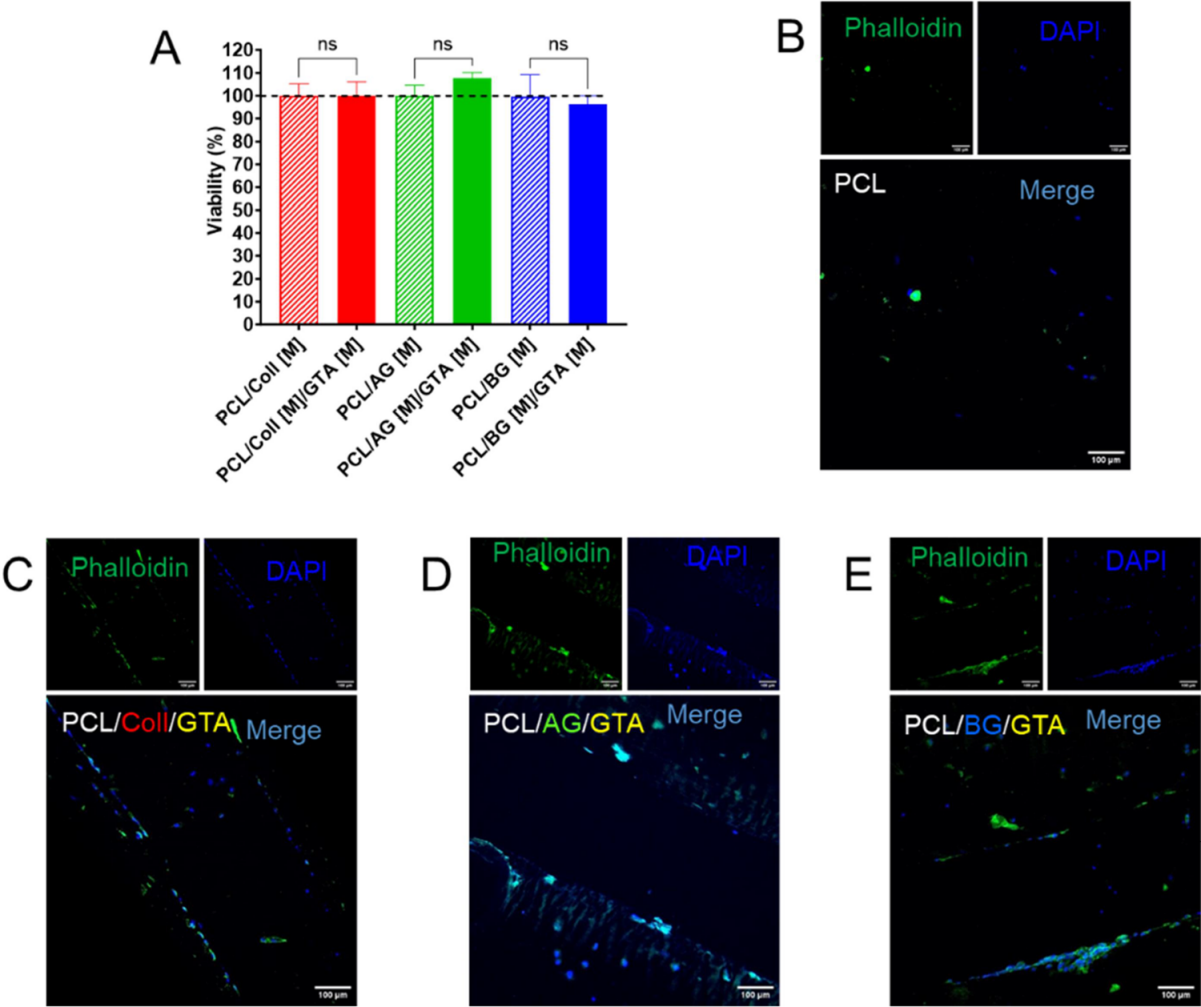
Natural polymer coatings are biocompatible and promote HEPM cell attachment to PCL scaffolds. (A) MTT assay of human embryonic palatal mesenchymal (HEPM) cells seeded on Coll-, AG-, or BG-coated PCL scaffolds with GTA (0.05 v/v%) crosslinking for 48 h. (B–E) Representative confocal images of DAPI (blue), fluorescein phalloidin (green), and their merged stained HEPM cells seeded on PCL scaffolds with GTA-crosslinked Coll C), AG (D), or BG (E) coating after 48 h.

The biocompatibility of the collagen, acidic gelatin, and basic gelatin coatings on 3D-printed PCL scaffolds with and without GTA crosslinking was assessed by measuring HEPM cell viability 48 h after being seeded on the different PCL scaffolds. We observed that the presence or absence of GTA crosslinking had no significant effect on cell viability after 48 h for all three coating materials (Figure 5A). We further assessed cell attachment to the different polymer coatings with GTA crosslinking using confocal microscopy (Figure 5B–E). HEPM cells were first seeded onto the differently coated PCL scaffolds with GTA crosslinking for 48 h, with noncoated PCL scaffolds as a control. The HEPM cells were then fixed and stained with nuclear DAPI and fluorescein phalloidin to visualize cell attachment and distribution on the differently coated PCL scaffolds. We found, while HEPM cells were able to attach to the noncoated PCL scaffold coatings to a small degree (Figure 5B), that HEPM cell attachment with positively stained cytoskeleton notably increased for all the groups in polymer-coated PCL scaffolds (Figure 5C–E). The attachment was also increased compared to non-GTA crosslinked polymer-coated PCL scaffolds (data no shown). Furthermore, we observed HEPM cell attachment in multiple layers of the PCL scaffold, penetrating the entire volume of the PCL structure, with the Coll- and BG-coated scaffolds exhibiting the most notable increase in the HEPM cell attachment and distribution.

### 3.4 Gelatin Type and Crosslinker Influence the Expression of *miR-200c* and Osteogenic Biomarkers

**Figure 6:**
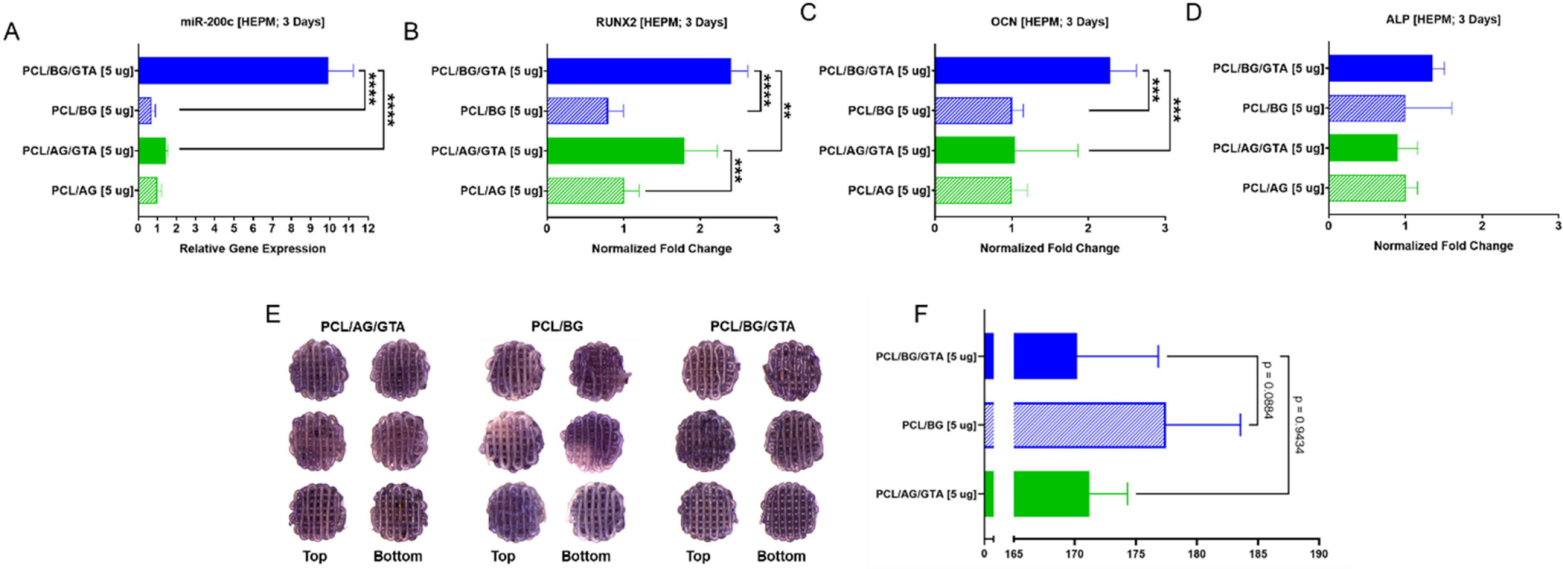
Glutaraldehyde (GTA) crosslinking of acidic (AG) or basic (BG) gelatin-coated PCL scaffolds incorporating pDNA *miR-200c* influences *miR-200c* overexpression and osteogenic differentiation in human embryonic palatal mesenchymal (HEPM) cells. (A) Relative expression levels of *miR-200c* from HEPM cells 3 days after seeding on AG- or BG-coated PCL scaffolds with or without GTA crosslinking. (B-D) Normalized fold change of *RUNX2* (B), *OCN* (C), and *ALP* (D) transcripts from HEPM cells 3 days after seeding on AG- or BG-coated PCL scaffolds with or without GTA crosslinking. (E, F) Top and bottom views (E) and gray color intensities (F) of ALP stained, HEPM-seeded scaffolds 14 days after seeding on AG- and BG-coated PCL scaffolds with GTA crosslinking and BG-coated scaffolds without crosslinking (*p* < 0.05; performed in triplicate).

HEPM cells were seeded onto *miR-200c*-incorporated acidic or basic gelatin-coated PCL scaffolds with or without GTA crosslinking for 3 days to evaluate how the release of *miR-200c* from the differently coated PCL scaffolds influenced *miR-200c* overexpression (Figure 6A) and the production of osteogenic biomarkers (Figure 6B–D) using qRT-PCR. We found that HEPM cells seeded on PCL scaffolds coated with GTA-crosslinked basic gelatin significantly increased *miR-200c* overexpression approximately 10-times higher than GTA-crosslinked acidic gelatin coatings and non-crosslinked basic gelatin coatings (Figure 6A). Furthermore, no significant differences in *miR-200c* overexpression were found between the acidic gelatin coatings with and without GTA crosslinking nor when comparing non-GTA crosslinked acidic and basic gelatin coatings. We also found that HEPM cells seeded on the GTA-crosslinked basic gelatin-coated PCL scaffolds significantly increased expression of *RUNX2* and *OCN* compared to acidic gelatin coatings with GTA crosslinking and non-crosslinked basic gelatin coatings (Figure 6B,C). There was also a statistically significant increase in *RUNX2* expression for the GTA-crosslinked acidic gelatin coatings in comparison to non-crosslinked acidic gelatin coatings; however, this increase was significantly less than that of the GTA-crosslinked basic gelatin coatings. We also observed an increase in *ALP* expression with the GTA-crosslinked basic gelatin-coated PCL scaffolds; however, this increase was not statistically different compared to the other coated scaffolds with or without GTA crosslinking (Figure 6D). Similarly, the activities of ALP were increased in the cells 14 days after seeding on GTA-crosslinked basic gelatin-coated PCL scaffolds compared to the other coated scaffolds with acidic gelatin and basic gelatin without GTA crosslinking (Figure 6E); however, this increase was not statistically different due to limited sample size (Figure 6F).

### 3.5 *miR-200c* Incorporated Gelatin Coatings on 3D-Printed PCL Scaffolds Enhance Calvarial Bone Formation *In Vivo*

**Figure 7:**
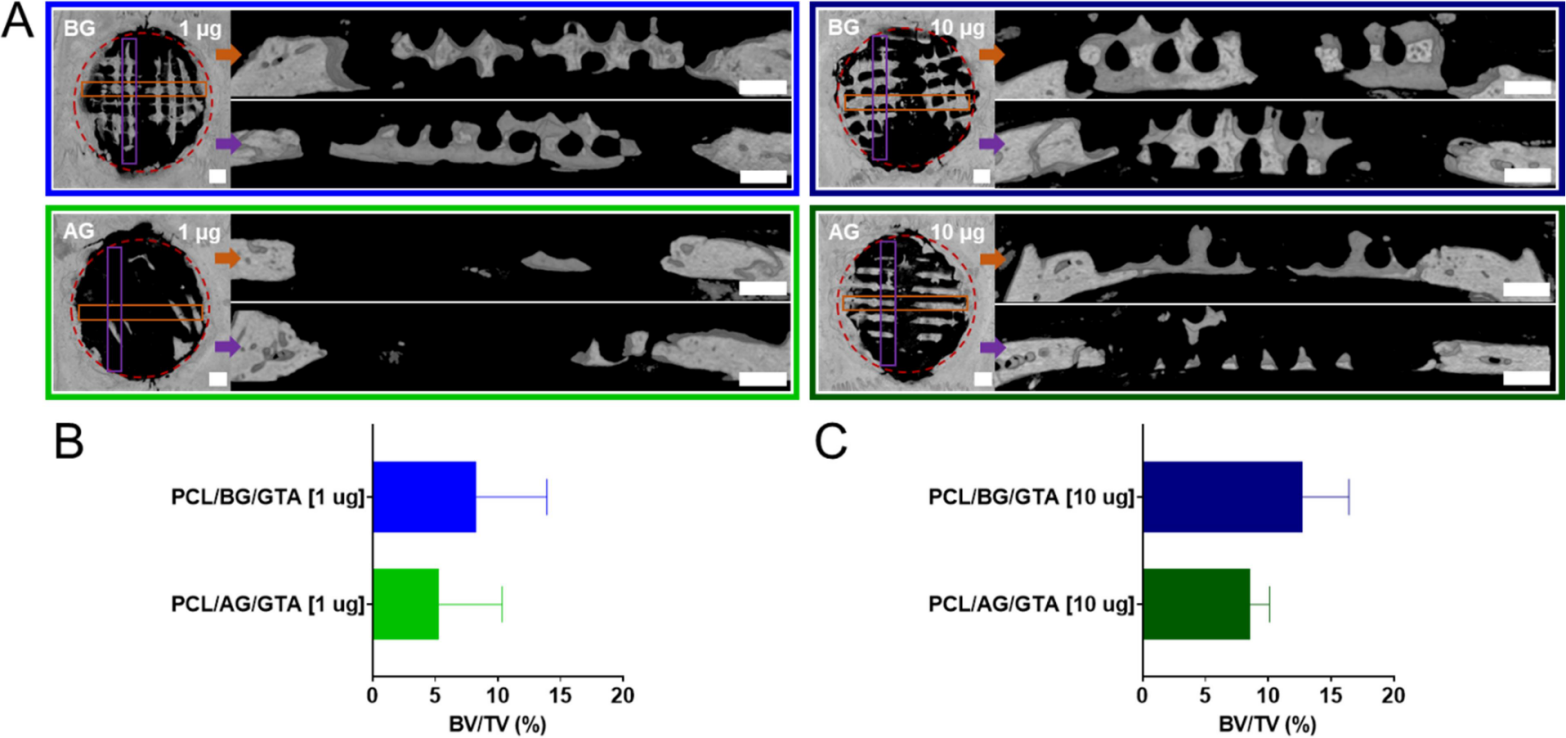
Microcomputed tomography (μCT) analysis of bone formation induced by *miR-200c*-incorporated PCL scaffolds coated with glutaraldehyde(GTA)-crosslinked acidic (AG) or basic (BG) gelatin implanted into critical-sized rat calvarial defects. (A) Representative μCT images of top and cross-sectional side views of explanted AG- or BG-coated PCL scaffolds incorporating *miR-200c* at 1 μg or 10 μg after 6 weeks of implantation. Cross-sectional images were taken across the diameter of the AG- or BG-coated PCL scaffolds in each direction (represented as orange or purple boxes) to assess bone regeneration within the 9 mm defect area containing the coated PCL scaffold (outlined as a red dashed circle). (B, C) Quantitative analysis of bone volume percentage in defects with PCL/(AG or BG)/GTA incorporating 1 μg *miR-200c* (B; *n* = 7/treatment) or 10 μg *miR-200c* (C; *n* = 3/treatment). Scale bars: 1 mm. BV: bone volume; TV: tissue volume.

The capacity for gelatin coatings to sustain the release of pDNA encoding *miR-200c* and enhance bone regeneration was evaluated *in vivo* using 9 mm diameter, critical-sized rat calvarial bone defects. Acidic or basic gelatin crosslinked with GTA was coated onto PCL scaffolds incorporating *miR-200c* [1 μg or 10 μg] and implanted into critical-sized rat calvarial defects for 6-weeks, after which bone formation in the defects was assessed via μCT imaging (Figure 7A) and bone volume percent quantification (Figure 7B,C). Through μCT imaging, we were able to visualize bone tissue growth occurring from the implanted coated PCL scaffolds (Figure 7A). We found that the acidic gelatin-coated PCL scaffolds incorporating *miR-200c* at 1 μg visually displayed less new bone formation compared to the basic gelatin-coated PCL scaffolds incorporating *miR-200c* at 1 μg. At the *miR-200c* 1 μg concentration, the basic gelatin-coated PCL scaffolds demonstrated an average new bone formation volume percentage of 8.29%, while the acidic gelatin-coated PCL scaffolds displayed a lower average bone volume percentage of 5.24% (Figure 7B). Visually, the basic gelatin-coated PCL scaffolds seem to produce more bone formation in the regenerating defect; however, the differences in bone volume percentage between the acidic and basic gelatin-coated PCL scaffolds incorporating *miR-200c* at 1 μg were not statistically significant due to large individual variations (*p* = 0.31). At the *miR-200c* 10 μg concentration, the basic gelatin-coated PCL scaffolds demonstrated an average new bone formation volume percentage of 12.71%, while the acidic gelatin-coated PCL scaffolds displayed a lower average bone volume percentage of 8.56%, and these differences in the average bone volume percentage between the acidic and basic gelatin-coated PCL scaffolds incorporating *miR-200c* at 10 μg were not statistically significant due to a small sample size (*p* = 0.24) (Figure 7C). However, by increasing the concentration of incorporated *miR-200c* from 1 to 10 μg, we found that we could increase bone formation occurring from both coating treatments—acidic gelatin-coated PCL (1 μg: 5.24%; 10 μg: 8.56%) and basic gelatin-coated PCL (1 μg: 8.29%; 10 μg: 12.71%).

**Figure 8:**
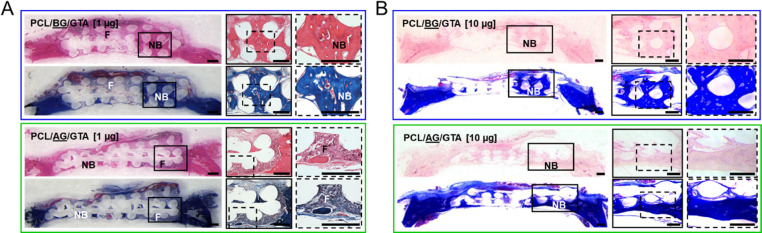
Histological analysis of new bone formation induced by *miR-200c*-incorporated PCL scaffolds coated with glutaraldehyde(GTA)-crosslinked acidic (AG) or basic (BG) gelatin implanted into critical-sized rat calvarial defects. (A, B) Microphotographs of the cross sections of critical-sized rat calvarial defects containing PCL/(AG or BG)/GTA scaffolds incorporating 1 and 10 μg *miR-200c* stained by H&E and Heidenhain’s azan trichrome at different magnifications. Scale bars: 500 μm. F: fibrous tissues; NB: new bone.

After μCT imaging and quantitative bone volume analysis, the explanted calvarial defects containing the differently coated PCL scaffolds were sectioned and stained to histologically examine new bone formation occurring within the implanted scaffolds. In the H&E and Heidenhain’s azan trichrome stained sections (Figure 8), we observed more fibrous tissue formation than bone formation occurring in the defects containing the acidic gelatin-coated PCL scaffolds incorporating *miR-200c* at 1 and 10 μg. The new bone tissues formed in the acidic gelatin-coated PCL scaffolds were found mainly in the lower, first layer of the implanted PCL scaffolds and in areas directly adjacent to the native calvarial bone. In the basic gelatin-coated PCL scaffold implants, however, we observed increased amounts of new bone formation occurring within multiple layers of PCL scaffolds. Additionally, within the areas of new bone tissue development in the basic gelatin-coated PCL scaffolds, we found evidence of new blood vessel formation typically associated with the formation of the new trabecular bone tissue.

## 4 Discussion

In this work, we aimed to create synthetic bone grafts that combine multiple biomaterials to structurally support bone defects and sustain the release of osteogenic miRs to enhance bone formation. Using fused deposition modeling, we 3D-printed PCL scaffolds with well-defined porous geometries and then coated them with collagen or gelatin, to increase scaffold hydrophilicity and cell attachment.^23−26^ These natural polymer coatings also functioned as a delivery system for the pDNA encoding osteoinductive *miR-200c*. To overcome the bulk release and rapid degradation properties of collagen hydrogel systems,^48−50^ this work leveraged the electrostatic properties of gelatin to enhance the bonding of incorporated pDNA encoding *miR-200c* to the gelatin polymer.^35−42^ Furthermore, by modulating the concentration of gelatin and the crosslinker, we were able to fine-tune our gelatin-coated PCL scaffolds to sustain the delivery of pDNA encoding *miR-200c* and improve regeneration in critical-sized bone defects by prolonging regenerative signaling.

### 4.1 Collagen- or Gelatin-Coating Effectively Increases Capabilities of PCL-Based Scaffolds for Tissue Engineering and Bone Regeneration

In tissue engineering applications, PCL is often used as a scaffold material as it is biocompatible, biodegradable, and easy to 3D print and has decent mechanical properties that can help support tissues as they regenerate. Yet, the hydrophobic nature of PCL can limit cell attachment, and combinations with other biologic materials, like natural polymer coatings like collagen or gelatin, are often needed to increase hydrophilicity, facilitate osseointegration, and promote osteogenesis. Therefore, we sought to utilize gelatin coatings on 3D-printed PCL scaffolds to drive increased cell attachment and osseointegration to PCL scaffolds while additionally providing a mechanism to extend the delivery of potent osteogenic biomolecules like pDNA encoding *miR-200c*. Using a multistep fabrication process (Figure 2), PCL scaffolds were first 3D-printed using FDM, then dip-coated in gelatin solution, and incorporated with pDNA encoding *miR-200c*. The 3D-printed PCL scaffolds were intentionally designed to include interconnected porous channels to support cell infiltration, growth, and nutrient and oxygen exchange.^51^ Furthermore, the PCL scaffolds had an average pore size of 450 μm (Table 3), which is within the 300–500 μm pore size range reported to induce osteogenic differentiation.^52,53^ Using this multistep, dip-coating method, we were able to effectively coat thin layers of gelatin onto 3D-printed PCL scaffolds, thus creating a multimaterial synthetic bone graft that encapsulated a delivery system to release incorporated pDNA encoding *miR-200c* for bone regeneration (Figure 3).

### 4.2 Natural Polymer Coating and Crosslinking Concentrations Influence pDNA Release from Coated PCL Scaffolds

In evaluating the capacity of our coated PCL scaffolds to improve cell attachment and sustain the delivery of incorporated pDNA molecules from PCL scaffolds, we created 3D-printed PCL scaffolds coated with different natural polymers (collagen, acidic gelatin, and basic gelatin) and assessed the release of incorporated pDNA over 72 h (Figure 4). Furthermore, natural polymers, like collagen and gelatin, are susceptible to rapid degradation by metalloproteinases like collagenase,^30^ but their mechanical strength and ability to retain incorporated biomolecules can be improved through chemical or physical crosslinking modifications.^54^ Crosslinking agents, including GTA, genipin, and others, have previously been utilized to improve gelatin delivery systems.^55^ By modulating the crosslinking content, gelatin coatings can be modified to slow release of complex agents to extend delivery. Therefore, using average concentrations from the literature,^35−42^ we evaluated how increasing coating material concentration influenced pDNA release as well as how different crosslinking agents, like GTA and genipin, altered pDNA release at increasing concentrations. The results of our release studies supported our initial hypothesis that the acidic, negatively charged gelatin would quickly release incorporated negatively charged pDNA, whereas the basic, positively charged gelatin would demonstrate slowed pDNA release profiles (Figure 1). Given our prior investigations on collagen as a carrier for pDNA encoding *miR-200c* in earlier studies, we included collagen as a comparative element in the release profile studies. From our pDNA release studies, we found that not only were there distinct differences in pDNA release profiles between the acidic and basic gelatins but we also found that our coated PCL scaffolds demonstrated release profiles comparable to commercially available natural polymer sponges (Figure 4A). Notably, the release profiles of pDNA encoding *miR-200c* from collagen are comparable to acidic gelatin but do not effectively sustain the release to the same extent observed with basic gelatin. Through our release studies, we also found that increasing the coating material concentration slowed pDNA release from all three polymer types (Figure 4B–D). This effect likely occurred because increasing the polymer concentration increased the polymer network packing density, which slowed coating degradation and impeded the release of incorporated pDNA molecules.^56,57^ We also found that GTA crosslinking decreased pDNA release for the collagen and acidic gelatin-coated PCL scaffolds, while the GTA-crosslinked basic gelatin-coated PCL scaffolds suspended pDNA release (Figure 4E). We further confirmed the significant influence of GTA concentration on the release of pDNA, particularly in basic gelatin, as opposed to acidic gelatin. Notably, while the low GTA concentration resulted in a release profile comparable to that of acid gelatin, the hydrogel with both medium and high concentrations sustained slower releases in basic gelatin than in acidic gelatin (Figure 4F–H). Additionally, we observed that a high GTA concentration led to hydrogel clotting within the porous structure of 3D-printed scaffolds, significantly affecting the pore size and cell migration. Consequently, we opted for a gelatin coating with a medium GTA concentration for further testing of scaffold functionality, incorporating *miR-200c* for osteogenic function assessment both in vitro and in vivo.

GTA is an effective crosslinker for gelatin but has been reported to be cytotoxic at high concentrations.^57−59^ Previous studies support that crosslinking with low-concentration GTA is nontoxic, and washing with glycine inactivates cytotoxic aldehyde groups in residual GTA.^46,60−63^ However, gelatin can also be crosslinked using genipin, which is slower and less efficient but less toxic crosslinker.^55,64−68^ From our release studies, we found that low concentrations of GTA can be used to effectively crosslink collagen- and gelatin-coated PCL scaffolds and alter pDNA release. However, genipin crosslinking was ineffective at reducing pDNA release, and all three coating materials crosslinked with genipin demonstrated burst release profiles where 80% of incorporated pDNA was released within 12 h (data not shown). The genipin crosslinking mechanism is much slower than that of GTA, and therefore, utilizing the same protocol used to crosslink our coatings with GTA likely did not allow genipin enough time to effectively create new bonds with the polymer network.^69−72^ Additional studies are required to thoroughly compare GTA and genipin crosslinking of polymer-coated scaffolds.

### 4.3 Crosslinking Gelatin Coatings on PCL Scaffolds Influence HEPM Cell Attachment

**Figure 9:**
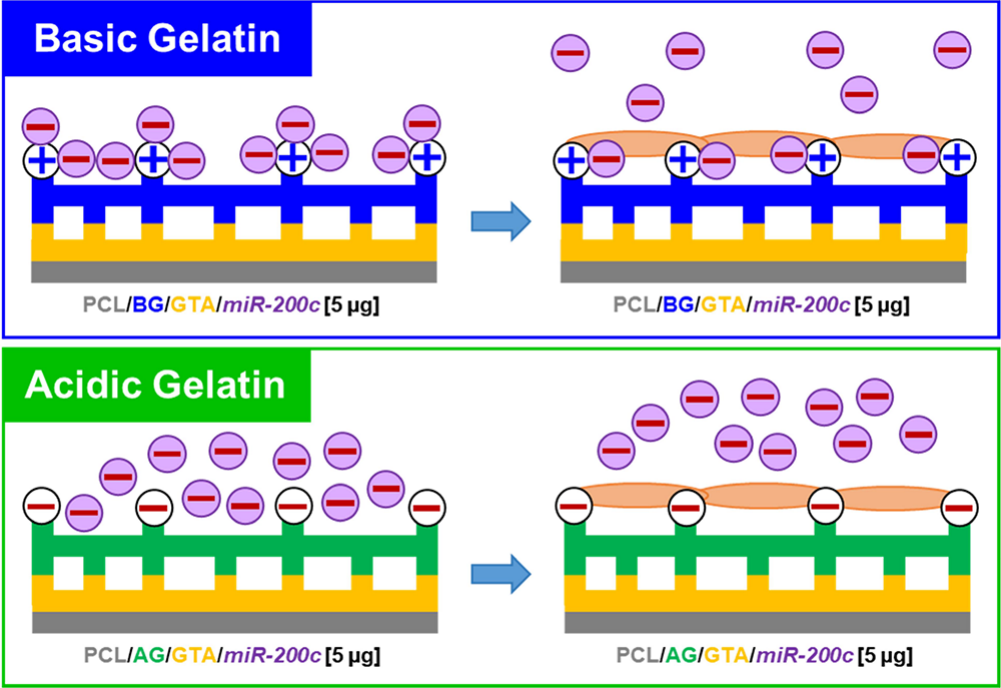
Illustration relating the release and uptake of pDNA encoding *miR-200c* from GTA-crosslinked gelatin-coated PCL scaffolds. Acidic gelatin coatings quickly release incorporated pDNA into the surrounding medium, and therefore, cells seeded on the acidic gelatin-coated scaffolds can only uptake pDNA encoding *miR-200c* from the medium. However, basic gelatin coatings slow pDNA release, and therefore, cells seeded on the basic gelatin-coated scaffolds can uptake pDNA encoding *miR-200c* released into the surrounding medium and directly from the pDNA-incorporated basic gelatin coating.

Our data also support that GTA crosslinking of gelatin coatings does not negatively affect cell bioactivity *in vitro* (Figure 5A). There were also no observable deleterious effects found in the *in vivo* implantation site with GTA-crosslinked gelatin-coated PCL scaffolds or within histologically stained explanted constructs, further supporting the notion that low-concentration GTA can be used as an effective crosslinker without cytotoxic effects. Using fluorescent confocal microscopy, we found that HEPM cells attached to PCL scaffolds coated with collagen, acidic, and basic gelatin and that GTA crosslinking of those coating materials slightly increased cell attachment (Figure 5B–E). More cells may have attached to the GTA-crosslinked collagen- or gelatin-coated PCL scaffolds because GTA crosslinking improved attachment of the polymer coating to the PCL scaffold. Early in developing our process to fabricate the polymer-coated PCL scaffolds, we found that we could improve attachment of the polymer solution to the PCL scaffold by first dip-coating with GTA solution. We were less successful using the inverse process, where scaffolds were first dip-coated in polymer and then GTA. In some of the non-crosslinked polymer-coated scaffolds, we observed cracks in the polymer coatings on the PCL scaffolds after lyophilization. The freeze-dried coatings without GTA crosslinking that experienced cracking of the polymer coating were likely more susceptible to portions of the coating flaking off during cell seeding or *in vitro* studies, which would expose the cells to noncoated, hydrophobic areas of the PCL filament and would likely decrease cell attachment. We did not observe this same cracking or flaking issue with the GTA-crosslinked polymer coatings, suggesting that GTA improved stability and attachment of the coating material to the PCL scaffold. This phenomenon may also explain the increase in *miR-200c* and osteogenic marker expression with GTA-crosslinked basic gelatin coatings (Figure 6). Release of pDNA encoding *miR-200c* was slowed by incorporation into basic gelatin-coated PCL due to the differences in electrostatic charge between the pDNA and basic gelatin (Figure 4), and with GTA crosslinking, cell attachment was increased (Figure 5). These two processes worked together to allow cells to attach to the coated PCL structure more readily and uptake incorporated pDNA from both the surface of the coating and pDNA released into the surrounding media. However, in the GTA-crosslinked acidic gelatin-coated PCL scaffolds, pDNA was rapidly released into the surrounding media, so the only source of pDNA for the cells was from the surrounding media and not the coating material. Figure 9 further illustrates the potential mechanisms governing the differences in pDNA release and cellular uptake observed between GTA-crosslinked acidic and basic gelatin-coated scaffolds. Furthermore, the increased cell attachment and stability of the polymer coatings via GTA crosslinking directed our decision to use GTA-crosslinked acidic or basic gelatin-coated PCL in our *in vivo* studies.

### 4.4 Basic Gelatin Coatings on PCL Scaffolds Improve *In Vivo* Bone Regeneration

We were unable to find statistically significant differences in the percent bone volume between the basic and acidic gelatin coatings in our study likely due to the relatively small number of animals and individual variations. Nevertheless, from our *in vivo* studies, we found that the basic gelatin coatings crosslinked with GTA visibly produced more bone regeneration than the acidic gelatin counterparts. We also found that we could further increase bone regeneration in both types of coated scaffolds by increasing the concentration of incorporated pDNA encoding *miR-200c* from 1 to 10 μg, thus demonstrating the regenerative effects of *miR-200c* as a potent osteoinductive agent (Figure 7A). At both *miR-200c* concentrations, the basic gelatin-coated scaffolds quantitatively increased bone regeneration in the defect space compared to acidic gelatin coatings (Figure 7B,C). From our histologically stained sections, however, we found more bone formation occurring in the defects treated with basic gelatin-coated PCL compared with the acidic gelatin-coated PCL (Figure 8). We additionally found that the acidic gelatin-coated PCL scaffolds showed bone formation mainly occurring in the first layer of the implanted scaffold, which was directly adjacent to the stem cell-incorporated periosteum layer in the calvaria. However, in the basic gelatin-coated PCL scaffolds, we observed bone formation occurring throughout the implanted scaffolds, not only in the bottom layer of the PCL scaffolds but also in the middle and upper layers of the scaffold. These data strongly support that sustained release of pDNA from gelatin-coated, 3D-printed PCL scaffolds potentially improves calvarial bone regeneration.

Future studies with larger sample sizes, multiple time points, and expanded substantial osteogenic biomarker measurements will be needed to evaluate the differences in regeneration potential between the two gelatin coating types and provide more details to relate the differences in pDNA release and bone regeneration potential between the acidic and basic gelatin coatings. Furthermore, our current *in vivo* studies utilized acidic or basic gelatin-coated PCL scaffolds crosslinked with GTA as we found GTA crosslinking increased cell attachment and coating stability on the PCL scaffolds *in vitro*. However, from our *in vitro* release studies, we observed a more significant difference in pDNA release between the non-crosslinked acidic and basic gelatin-coated PCL scaffolds than those crosslinked with GTA. Therefore, future *in vivo* studies utilizing non-crosslinked acidic or basic gelatin-coated PCL scaffolds may demonstrate more significant differences in bone regeneration; however, processes to improve coating attachment to the PCL without GTA would need to be improved prior to these studies.

The results of this study demonstrate that multiple biomaterials can be combined to create a synthetic bone graft with drug delivery capabilities and that by modulating polymer coating concentration and crosslinking, we can alter pDNA release to influence bone regeneration. Our data further demonstrate that the electrostatic charge differences between acidic and basic gelatin polymers influence retention and release of negatively charged pDNA molecules. This study also provides additional evidence to support *miR-200c* as a potent osteoinductive biomolecule capable of enhancing osteogenic differentiation and bone formation. The novel combination of 3D-printed PCL scaffolds with gelatin coatings incorporating pDNA encoding *miR-200c* provided us with a synthetic bone graft capable of enhancing bone regeneration, and the results of our study further illustrate the potential therapeutic application of miR-incorporated tissue-engineered synthetic bone grafts for regenerating large bone defects.

## 5 Conclusions

There is a critical need to advance the development of synthetic bone grafts toward clinical application for treating large bone defects. Using a novel, multimaterial scaffold fabrication approach that combined 3D-printed PCL scaffolds with natural polymer coatings, we created a synthetic construct with drug delivery capabilities applicable to bone tissue engineering and regeneration. By incorporating pDNA encoding *miR-200c* into gelatin coatings on PCL scaffolds, we demonstrated that we could effectively enhance osteogenic differentiation *in vitro* and bone formation *in vivo*. Furthermore, for the first time, we effectively demonstrated that the release of pDNA encoding *miR-200c* from gelatin coatings on 3D-printed PCL scaffolds was dependent on gelatin type, coating material concentration, and crosslinking. These data further illustrate the potential use of basic gelatin materials in the delivery of pDNA molecules for gene therapy and tissue engineering applications. Moreover, the release profiles of pDNA encoding *miR-200c* from gelatin-coated scaffolds and the effects of modulating coating material properties on *in vitro* release rate and *in vivo* bone formation had not previously been evaluated prior to this study. Our results effectively demonstrate the potential of miR-incorporated synthetic bone grafts to enhance bone regeneration, and further optimization of gelatin-based delivery systems that sustain the release of pDNA encoding osteoinductive miRs is needed in the development of efficient synthetic grafts that maximize bone regeneration to restore large bone defects.
